# Analysis of serum polyunsaturated fatty acid metabolites in allergic bronchopulmonary aspergillosis

**DOI:** 10.1186/s12931-020-01471-4

**Published:** 2020-08-05

**Authors:** Lu Li, Jianlin Wu, Xiqing Bian, Ge Wu, Peiyan Zheng, Mingshan Xue, Baoqing Sun

**Affiliations:** 1 Department of Allergy and Clinical Immunology, State Key Laboratory of Respiratory Disease, National Clinical Research Center of Respiratory Disease, Guangzhou Institute of Respiratory Health, First Affiliated Hospital of Guangzhou Medical University, Guangzhou, Guangdong China; 2 Sino-French Hoffmann Institute of Immunology, Guangzhou Medical University, Guangzhou, Guangdong China; 3 State Key Laboratory of Quality Research in Chinese Medicine, Macau Institute for Applied Research in Medicine and Health, Macau University of Science and Technology, Taipa, Macao China

**Keywords:** ABPA, HETEs, Lipid mediators, Polyunsaturated fatty acid

## Abstract

**Background:**

The importance of lipid mediators in allergic diseases has been long recognized, whereas little is known about their role in allergic bronchopulmonary aspergillosis (ABPA). We investigated whether lipid mediators are associated with ABPA.

**Methods:**

We recruited 12 ABPA patients, 23 asthma patients and 12 healthy control in our study. Serum of 11 ABPA patients were collected before and following treatment. 36 polyunsaturated fatty acid metabolites were measured in serum samples by using liquid chromatography–mass spectrometry. This study was approved by the Ethics Committee of the First Affiliated Hospital of Guangzhou Medical University, with ethics number GYFYY-2016-73.

**Results:**

Levels of arachidonic acid (AA), 15(S)-hydroxyeicosatetraenoic acid (HETE), 12(S)-HETE, 8(S)-HETE, 5(S)-HETE, LTB_4_, PGB_2_, 12(S)-hydroxyeicosapentaenoic acid (HEPE), 12-hydro-xyheptadecatrienoic acid (HHTrE) were significantly higher in ABPA patients than that in HC groups. Compared with asthma group, ABPA group expressed lower levels of 15(S)-hy-droperoxyeicosatetraenoic acid (HPETE), 5(S)-HPETE, 13(S)-hydroperoxyoctadecadienoic acid (HPODE) and 9(S)-HPODE. In APBA patients, AA level was positively correlated with serumtotal IgE (tIgE). The levels of 12(S)-HPETE, 15(S)-HEPE and 12(S)-HEPE correlated with *Asp-ergillus fumigatus* specific IgE(*A. fumigatus* sIgE) positively. Peripheral blood eosinophilia correlated with high levels of 12(S)-HETE and 15(S)-HETE. In addition, the serum levels of15(S)-HETE and 12(S)-HETE in ABPA subjects both declined with the decrease of tIgE, *A. fumigatus* sIgE and sIgG concentrations after treatment.

**Conclusions:**

We present data regarding the role of polyunsaturated fatty acid metabolites in APBA for the first time. Most of the tested metabolites increased in ABPA when co-mpared with healthy controls and 15(S)-HETE and 12(S)-HETE may play a role in the pat-hogenesis of ABPA. These findings can provide new ideas for diagnosis, therapy and mon-itor of ABPA.

## Background

Allergic bronchopulmonary aspergillosis (ABPA) is a complex pulmonary disorder that results from immune hypersensitivity to *Aspergillus fumigatus (A. fumigatus)* colonizing the airways, which is common among patients with asthma and cystic fibrosis [1, 2]. It is characterized by a Th2 bias immune response, peripheral blood and pulmonary eosinophilia, increased total serum IgE, increased *A. fumigatus* sIgE and *s*IgG [2, 3]. The pathogenesis of ABPA remains unclear. By 2013, there are estimated 4.8 million ABPA patients worldwide and the prevalence of ABPA in adults with asthma was 2.5% (range 0.72–3.5%) [4]. However, due to diverse and atypical clinical manifestations, ABPA is easily misdiagnosed and missed by clinicians [5]. There are also limited drugs for the treatment of ABPA. Glucocorticoids and antifungal agents are mainly used clinically, which is associated with numerous side effects [2]. Therefore, it is crucial to identify specific mechanisms and therapies in ABPA.

Over the last few decades, the role of lipid mediators on allergic inflammation gained increasing clinical attention. The arachidonic acid (AA) metabolites are becoming increasingly important in the mechanisms underlying the inflammatory reaction associated to asthma [6–8]. Eicosanoids, including 5-Lipoxygenase (5-LOX) metabolites leukotrienes (LTs) and cyclooxygenase (COX) products prostaglandins (PGs), are well known lipid metabolites involving in asthma [9]. 12/15-LOX are associated with the allergen-induced airway inflammation in mouse allergic model [10]. And plasma 15-hydroxyeicosatetraenoic acid (15-HETE), derived from AA via the 15-LOX pathway, is reported to predict the treatment outcomes in aspirin-exacerbated respiratory disease [11]. In addition, one approved 5-LOX inhibitor, zileuton, has been found as a therapy for asthma, demonstrating the importance of lipid mediators in human allergic disease [6, 12, 13].

The pathogenesis of ABPA largely remains speculative [2, 14] and exploring the role of the lipid inflammatory mediators in ABPA may provide new ideas for diagnosis and therapy of ABPA. Therefore, the aim of this study was to compare serum lipid mediators of ABPA and asthma and to investigate correlation of lipid mediator and clinical parameters in ABPA patients. We use mass spectrometry to measure 36 lipid metabolite levels in serum from patients with ABPA, asthma and healthy controls and observed the changes of lipid metabolites during the treatment of ABPA patients.

## Methods

### Subjects

This is a retrospective study by using data from the Allergy Information Repository of State Key Laboratory of Respiratory Disease in China. We enrolled 12 patients with ABPA, 23 patients with allergic asthma and 12 healthy controls from the First Affiliated Hospital of Guangzhou Medical University. The diagnosis of ABPA was based on the criteria of the International Society for Human and Animal Mycology (ISHAM) working Group [15]. The diagnosis of asthma was based on clinical history and medical examination, spirometry and FEV1 reversibility≥12% demonstrated at least once in the previous 6 months according to Global Initiative for Asthma (GINA). The healthy controls were screened from physical examination volunteers in our hospital. Their routine physical examination results were all within the normal range and none had a history of asthma or allergy. All subjects studied gave informed consent to participate. Serum samples of 11 ABPA patients were collected prior to and at 1, 2, 3 and 6 months after treatment. All of them had received standard therapy using oral corticosteroids prednisone acetate (15-30 mg/d) and antifungal agents itraconazole (400-500 mg/d) with other symptomatic support therapy. All asthma samples were collected before hospitalization and treatment. No patient had an aspirin-exacerbated respiratory disease (AERD) in this study.

### Blood collection, serum processing and storage

Blood samples were collected in coagulation tube with separation gel (Becton Dickinson, New Jersey, United States of America) and centrifuged for 10 min at 3000 rpm for the preparation of serum. In order to avoid repeated freezing and thawing, the remaining serum samples were kept in a − 80° Celsius refrigerator for following tests.

### Detection of IgE

The serum total IgE level and *A. fumigatus*-sIgE level were detected by using an ImmunoCap1000 system (Thermo Fisher Scientific Inc., California, USA). A test was considered positive when total IgE > 60 IU/mL, sIgE≥0.35 kU/L were considered positive.

### UHPLC-Q-TOF/MS analysis of lipid mediators in serum

Analyses were performed with an Agilent 1290 Infinity LC system (UHPLC, Santa Clara, CA) coupled to an Agilent 6550 UHD accurate-mass Q-TOF/MS system with a dual Jet stream electrospray ion source (dual AJS ESI). Raw data extraction was performed using Mass Hunter qualitative analysis software (Agilent Technologies) and then the date were performed with Mass Profiler Professional (MPP) software (Agilent Technologies). The metabolites were identified based on the standards, MS/MS spectra, and the metabolites database Lipid Maps (http://www.lipidmaps.org/) and METLIN (https://metlin.scripps.edu/index.php).

### Statistical analysis

Statistical analysis was performed using SPSS version 18 (SPSS Inc., Chicago, IL, USA) and GraphPad Prism 5.0 software (GraphPad Software, San Diego, CA, USA). Data are presented as the median with 25–75 interquartile range. Kruskal–Wallis H test for non-parametric variables are used to compare the difference of lipid mediator levels among the three groups. Correlation analyses were performed by Pearson’s test or Spearman’s test as appropriate with respect to data distribution. Statistical significance was set at *p* < 0.05. The heatmap was performed using the OmicShare tools, a free online platform for data analysis (http://www.omicshare.com/tools).

## Results

### Clinical characteristics

The clinical characteristics of recruited subjects are presented in Table 1. We studied 12 patients with ABPA, 23 patients with asthma and 9 healthy controls. These three groups did not differ by age and gender. Furthermore, there was no significant difference in lung function between ABPA and asthma groups. Most of the subjects had no smoking history, but one of the patients with ABPA had smoked for 19 years with 1 pack per day and three of patients with asthma had smoked for at least 10 years with at least 1 pack per day. Compared with healthy subjects, the ABPA and asthma patients exhibited significantly higher levels of peripheral blood eosinophil count, serum total IgE and *Af.* sIgE. Furthermore, patients with ABPA had the highest serum total IgE and *A. fumigatus* sIgE levels among these three groups. In addition, 3 individuals (25.00%) of ABPA group had taken oral glucocorticoids (eg. prednisone acetate) by themselves before the first sampling. Five asthma patients (21.74%) had taken inhaled glucocorticoids (eg. symbicort) at home before sampling.

**Table 1:** Clinical characteristics of all subjects participating in this study

	ABPA (***N*** = 12)	Asthma (***N*** = 23)	HC (*N* = 12)
**Sex (male/female)**	7/5	11/12	5/7
**Age (years)**	48 (28–52)	35 (20–48)	41 (38–44)
**Smoker/non-smoker**	1/11	3/20	0/12
**FVC (% pred)**	86.80 (69.10–102.10)	96.20 (87.55–101.40)	NA
**FEV1 (% pred)**	72.00 (54.60–87.80)	87.30 (76.70–94.80)	NA
**FEV1/FVC (%)**	88.60 (81.10–97.90)	78.30 (69.60–83.50)	NA
**FEF25–75%pred**	54.60 (24.90–72.10)	55.00 (42.80–69.90)	NA
**Sputum Eosinophils (%)**	12.50 (6.05–40.00)	7.75 (0.75–21.13)	NA
**Blood Eosinophils (%)**	10.60 (7.00–21.45)	3.75 (2.93–4.58)	2.37 (1.33–3.33)**
Blood Eosinophils (*10^**9**^**/l)**	0.92 (0.49–1.46)	0.22 (0.18–0.32)	0.15 (0.10–0.20)***^□□^
**TIgE (kU/l)**	2681.00 (1987.00–5000.00)	504.00 (147.00–735.00)**	42.47 (14.90–60.83)***^□□^
***Af.*sIgE (kU/l)**	9.70 (2.57–21.55)	0.07 (0.04–0.72)**	0.02 (0.00–0.02)***^□□^
**Using glucocorticoids/not**	3/9	5/18	0/12

Values are presented as medians [25–75 interquartile range]

* *P*-values compared with ABPA patients: *P < 0.05, **P < 0.01, ***P < 0.001

^□^*P*-values compared with asthma patients: ^□^*P* < 0.05, ^□□^*P* < 0.01, ^□□□^*P* < 0.001

*ABPA* Allergic bronchopulmonary aspergillosis, *HC* Healthy controls, *FVC* Forced vital capacity, *FEV1* Forced expiratory volume in one second, *FEF25–75%* Forced expiratory flow from 25 to 75% of FVC, *TIgE* Total IgE, *Af. Aspergillus fumigatus, NA* Not available

### Comparison of serum lipid mediator levels in ABPA, asthma and healthy controls

**Fig. 1:**
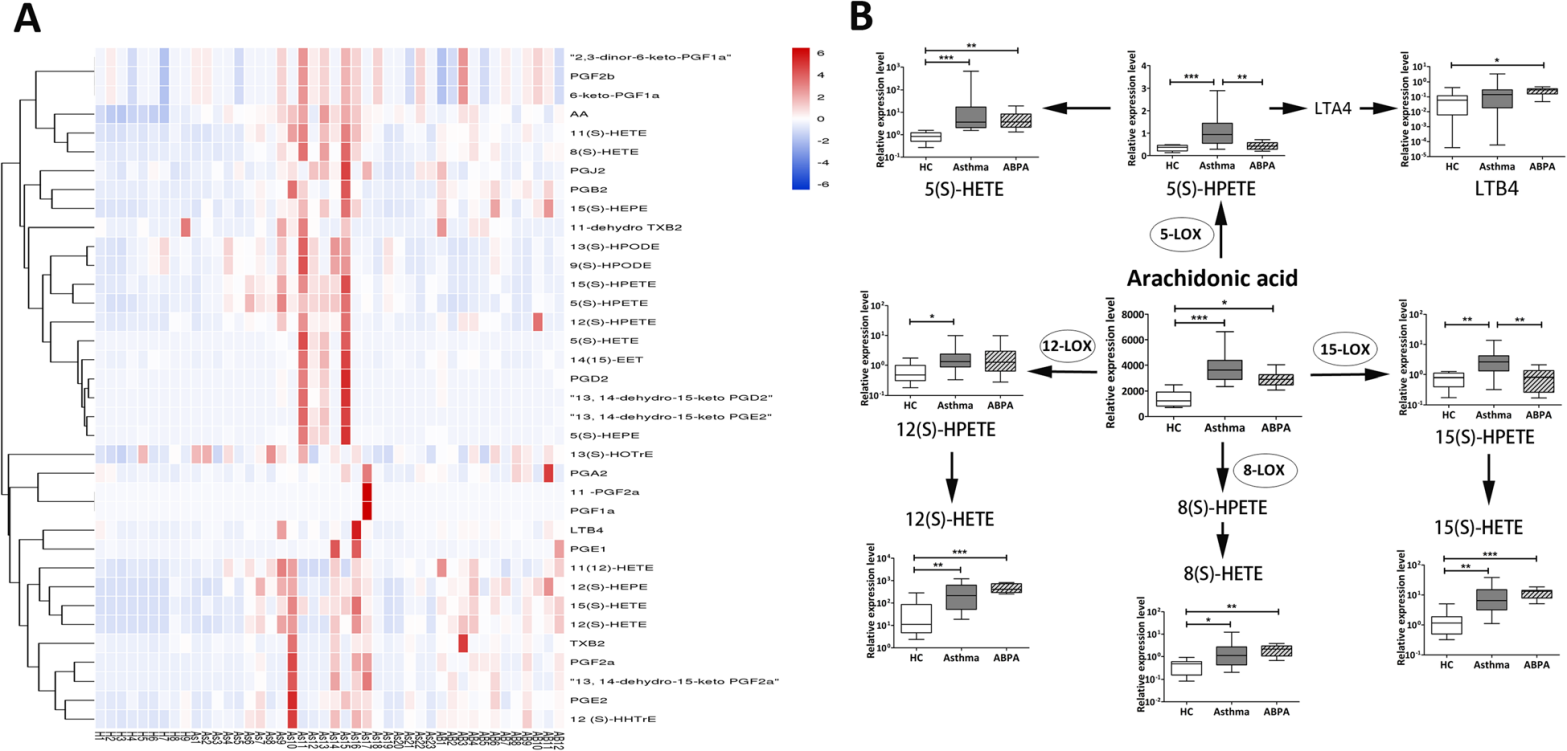
Comparison of serum lipid metabolites in ABPA, asthma and healthy control. **a** Heatmap analysis of all tested lipid mediators. H, healthy control; As, Asthma; AB, ABPA. **b** Comparison of serum eicosanoids from the lipoxygenase pathway**.** Center values indicate medians. Statistical significance was calculated by using the Mann–Whitney U test.*P < 0.05, **P < 0.01, ***P < 0.001. ABPA, allergic bronchopulmonary aspergillosis; HC, healthy controls; HPETE, hydroperoxyeicosatetraenoic acid; HETE, hydroxyeicosatetraenoic acid; LTB4, leukotriene B4; LOX, Lipoxygenase

We measured 36 lipid mediators in serum and they were analyzed using heatmap (Fig. 1a). Most of them are arachidonic acid (AA) metabolites. As show in Fig. 1a, the lipid mediators levels in healthy control group were very low and the asthma group seem to had the highest levels. Different metabolite levels showed strong difference. Both asthmatic subjects (3619.61; [2926.52–4309.04]) and ABPA patients (3018.84; [2596.09–3296.18]) had significantly higher concentrations of AA than that in healthy controls (Fig. 1b, *P* < 0.001 and *P* < 0.05). Likewise, the metabolites 15(S)-HETE, 12(S)-HETE, 8(S)-HETE and 5(S)-HETE derived from the LOX activity on AA showed similar trend (Fig. 1b, *p* < 0.05). Asthma patients had significantly higher levels of 15(S)-HPETE and 5(S)-HPETE when compared with ABPA group and healthy controls (Fig. 1b, *P* < 0.01). In addition, the other lipid mediator levels were listed in Table 2. The metabolites of linoleic acid, 9- and 13-hydroperoxylinoeic acid [9(S)-HPODE and 13(S)-HPODE], were significantly higher in asthmatic subjects when compared with ABPA group and healthy controls (Table 2, *P* < 0.01).

**Table 2:** lipid metabolite levels in serum of ABPA, asthmatic patients and healthy control groups

	ABPA (N = 12)		Asthma (N = 23)		HC (N = 12)	
AA	3018.84	[2596.09–3296.18]	3619.61	[2926.52–4309.04]	1232.41	[889.29–1872.78] *^□□□^
15(S)-HPETE	0.84	[0.54–1.26]	2.62	[1.45–4.14] **	0.80	[0.46–1.06] ^□□^
12(S)-HPETE	0.89	[0.26–3.62]	1.33	[0.89–2.34]	0.47	[0.25–1.07] ^□^
5(S)-HPETE	0.43	[0.35–0.55]	0.94	[0.56–1.37] **	0.37	[0.23–0.44] ^□□^
15(S)-HETE	11.56	[8.06–14.20]	6.50	[3.52–14.27]	1.16	[0.38–2.65] ***^□□^
12(S)-HETE	367.36	[290.68–546.39]	214.22	[54.38–552.75]	11.10	[17.54–128.41] ***^□□^
11(S)-HETE	3.38	[2.79–4.54]	3.28	[1.38–6.24]	0.80	[0.44–1.57] **^□□^
8(S)-HETE	1.83	[1.25–2.68]	1.13	[0.51–2.32]	0.48	[0.22–0.64] **^□^
5(S)-HETE	2.98	[2.06–8.89]	3.65	[2.09–14.29]	0.84	[0.55–1.19] ** ^□□□^
LTB4	0.22	[0.16–0.33]	0.14	[0.03–0.28]	0.06	[0.01–0.19]*
15(S)-HEPE	0.27	[0.19–0.71]	0.19	[0.08–0.41]	0.07	[0.03–0.11]
12(S)-HEPE	5.48	[3.27–8.40]	2.85	[1.01–6.05]	0.18	[0.18–13.63]*** ^□□^
5(S)-HEPE	0.36	[0.19–1.02]	0.68	[0.44–1.67]	0.16	[0.05–0.48]
9(S)-HPODE	5.31	[4.27–9.47]	12.58	[7.35–25.32]*	7.16	[3.83–9.37]
13(S)-HPODE	5.38	[4.26–9.51]	12.7	[6.71–25.99]*	7.61	[3.75–9.66]
PGB2	0.17	[0.08–0.24]	0.04	[0.01–0.13]	0.18	[0.09–0.37]***^□^
12HHTrE	86.00	[63.40–91.69]	47.20	[27.55–86.35]	3.70	[2.15–17.03]***^□^
11 (12)-HETE	0.67	[0.44–1.34]	0.64	[0.41–1.24]	0.23	[0.17–0.35]** ^□□^

Values are presented as medians [25–75 interquartile range]

* *P*-values compared with ABPA patients: *P < 0.05, **P < 0.01, ***P < 0.001

^□^*P*-values compared with asthma patients: ^□^*P* < 0.05, ^□□^*P* < 0.01, ^□□□^*P* < 0.001

*ABPA* Allergic bronchopulmonary aspergillosis, *HC* Healthy controls, *AA* Arachidonic acid, *HPETE* Hydroperoxyeicosatetraenoic Acid, *HETE* Hydroxyeicosatetraenoic acid, *LTB4* Leukotriene B4, *HEPE* Hydroxyeicosapentaenoic acid, *HPODE* Hydroperoxyoctadecadienoic acid, *PGB2* Prostaglandin B2, *HHTrE* Hydroxyheptadecatrienoic acid

### Correlation between lipid mediator levels and clinical parameters in ABPA

**Fig. 2:**
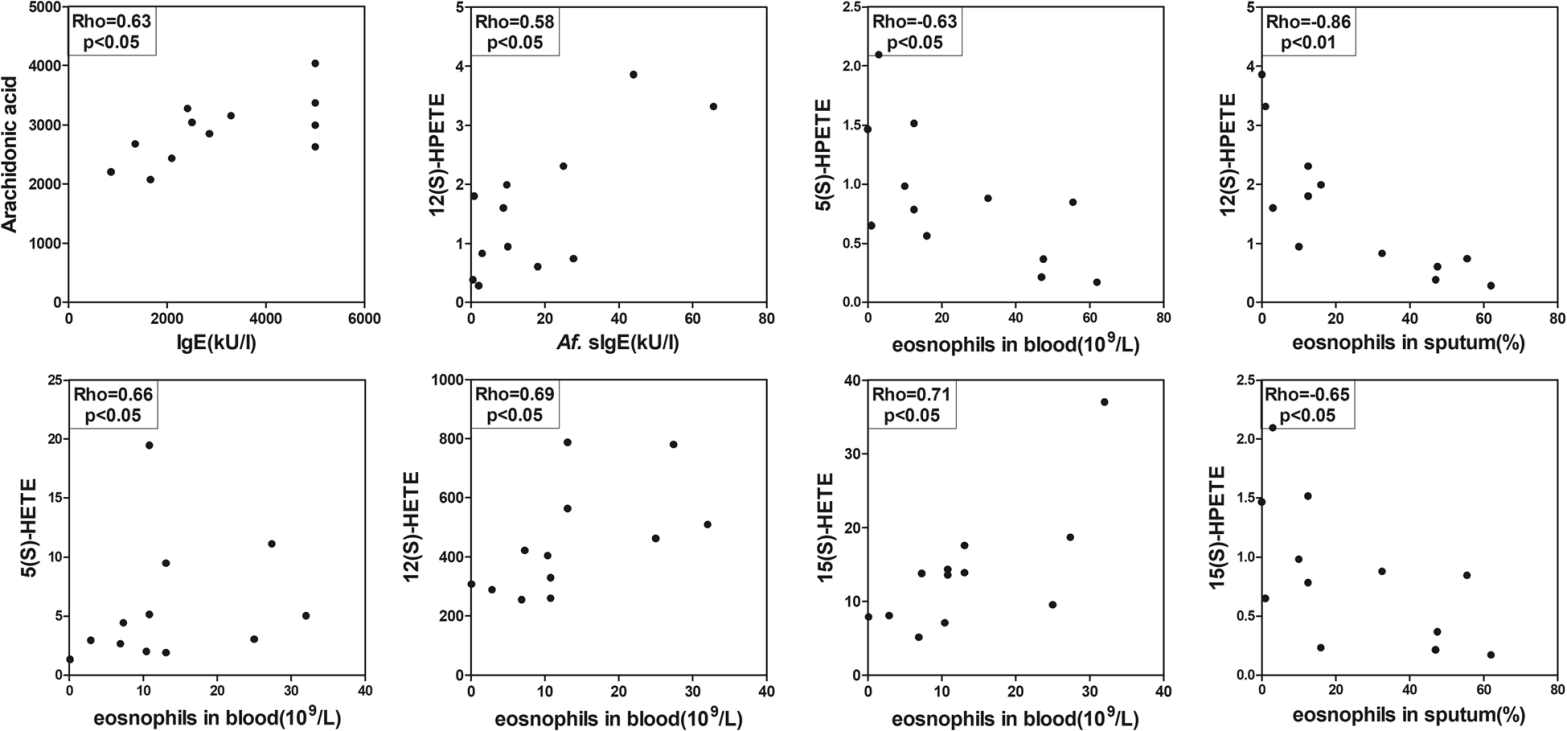
Correlation between serum eicosanoids from the LOX pathway and specific clinical parameters in ABPA. Statistical analysis was performed with the Spearman rank test. HPETE, hydroperoxyeicosatetraenoic acid; HETE, hydroxyeicosatetraenoic acid

The possible clinical significance of these lipid mediators in ABPA was evaluated by correlation analysis. AA and its metabolites from the LOX pathway showed significant correlation with the ABPA specific clinical parameters. As show in Fig. 2, serum AA level was positively correlated with the total IgE level (R = 0.63; *P* < 0.05). Moreover, 12(S)-HPETE was correlated with *A. fumigatus* sIgE level (R = 0.58, *P* < 0.05). It is noted that, all 3 LOX products, 5(S)-HETE, 12(S)-HETE and 15(S)-HETE showed significant positive correlation with the peripheral blood eosinophil count (Fig. 2, R = 0.66, 0.69, 0.71 respectively, *p* < 0.05). However, the precursor 5(S)-HPETE correlated negatively with the peripheral blood eosinophil count (R = -0.63, *p* < 0.05) while 12(S)-HPETE and 15(S)-HPETE showed significant negative correlation with the percentage of sputum eosinophils (R = -0.86, *p* < 0.01 and R = -0.65, *P* < 0.05).

Correlation analysis of other lipid mediator and clinical parameters are shown in Table 3. In addition to AA metabolite, the eicosapentaenoic acid (EPA) metabolites also showed significant correlation with ABPA clinical parameters. 15- and 12- hydroxyeicosapentaenoic acid [15(s)-HEPE and 12(S)-HEPE] were positively correlated with the *A. fumigatus* sIgE level (R = 0.59, *p* < 0.05 and R = 0.73, *p* < 0.01). 13(S)-HPODE and 9(S)-HPODE were negatively correlated with the percentage of sputum eosinophils (R = -0.68, *p* < 0.05 and R = -0.70, *p* < 0.05). The cyclooxygenase (COX) product 12-hydroxyheptadecatrienoic acid (12-HHTrE) showed positive correlation with peripheral blood eosinophil count and %FEV1 (R = 0.62, *p* < 0.05 and R = 0.70, *p* < 0.05).

**Table 3:** Correlation analysis of lipid mediator levels and clinical parameters in ABPA

Rho	AA	15(S)- HPETE	12(S)- HPETE	5(S)- HPETE	15(S)- HETE	12(S)- HETE	11(S)- HETE	8(S)- HETE	5(S)- HETE	LTB4	15(S)- HEPE	12(S)- HEPE	5(S)- HEPE	13(S)- HPODE	9(S)- HPODE	12 HHTrE	PGD2	PGB2
**tIgE (kU/l)**	0.63*	0.15	0.33	0.37	0.14	0.33	0.41	0.26	− 0.08	− 0.51	− 0.31	0.11	− 0.29	0.29	0.24	0.32	− 0.15	− 0.20
**Af. sIgE (kU/l)**	0.25	0.36	0.58*	0.29	0.23	0.40	0.33	0.32	0.06	0.11	0.59*	0.73**	0.44	0.20	0.24	0.05	0.42	−0.03
**NEU in Blood(10**^**9**^**/L)**	0.36	0.22	0.45	0.18	0.06	0.15	0.53	0.30	−0.03	− 0.38	− 0.21	0.04	− 0.17	0.26	0.27	0.43	0.08	0.13
**NEU in Blood (%)**	−0.23	0.26	0.68*	0.30	0.22	0.10	−0.01	0.13	0.01	0.15	0.58*	0.24	0.30	0.06	0.10	−0.27	0.37	−0.05
**EOS in Blood (10**^**9**^**/L)**	0.41	−0.25	−0.13	− 0.63*	0.71*	0.69*	0.55	0.31	0.66*	0.43	0.07	−0.14	0.38	−0.39	−0.42	0.62*	0.28	−0.13
**EOS in Blood (%)**	0.41	−0.30	−0.17	− 0.59*	0.78**	0.74**	0.56	0.28	0.56	0.45	0.16	−0.04	0.38	−0.46	−0.49	0.61*	0.24	−0.09
**NEU in Sputum (%)**	0.41	0.32	0.22	−0.20	0.64*	0.32	0.43	0.46	0.55	0.65*	0.45	0.19	0.77**	0.13	0.14	0.30	0.24	0.01
**EOS in Sputum (%)**	−0.42	−0.65*	−0.86**	−0.45	0.03	0.20	−0.11	−0.19	0.00	0.10	−0.14	−0.22	− 0.23	−0.68*	**− 0.70**	0.08	− 0.12	0.38
**LYM in Sputum (%)**	0.00	−0.48	−0.44	− 0.24	−0.17	− 0.23	0.14	− 0.06	−0.23	− 0.32	−0.68*	− 0.49	−0.64*	− 0.28	−0.30	0.38	−0.73**	0.30
**%FEV1**	0.58*	0.35	0.19	−0.17	0.37	0.32	0.80**	0.40	0.16	−0.06	0.23	0.26	0.41	0.22	0.25	0.70*	0.22	0.20
**FEV1/FVC (%)**	0.23	−0.36	−0.20	−0.32	0.09	0.24	−0.02	**−0.65**	− 0.28	−0.24	− 0.18	−0.23	− 0.21	−0.34	− 0.36	0.19	0.35	−0.54
**%FEF25–75**	0.67*	−0.06	0.19	−0.16	0.66*	0.62*	0.63*	0.15	0.05	0.12	0.30	0.35	0.29	−0.15	−0.17	0.58*	0.18	−0.20

Correlation analyses were performed using Spearman rank test. *P < 0.05, **P < 0.01

*TIgE* Total IgE, *Af.****Aspergillus fumigatus****, NEU* Neutrophils, *EOS* Eosinophils, *LYM* Lymphocytes, *FVC* Forced vital capacity, *FEV1* Forced expiratory volume in one second, *FEF25–75%* Forced expiratory flow from 25 to 75% of FVC, *AA* Arachidonic acid, *HPETE* Hydroperoxyeicosatetraenoic acid, *HETE* Hydroxyeicosatetraenoic acid, *LTB4* Leukotriene B4, *HEPE* Hydroxyeicosapentaenoic acid, *HPODE* Hydroperoxyoctadecadienoic acid, *PGB2* Prostaglandin B2, *HHTrE* Hydroxyheptadecatrienoic acid

### Baseline and post-treatment values of arachidonic acid metabolites in ABPA patients

**Fig. 3:**
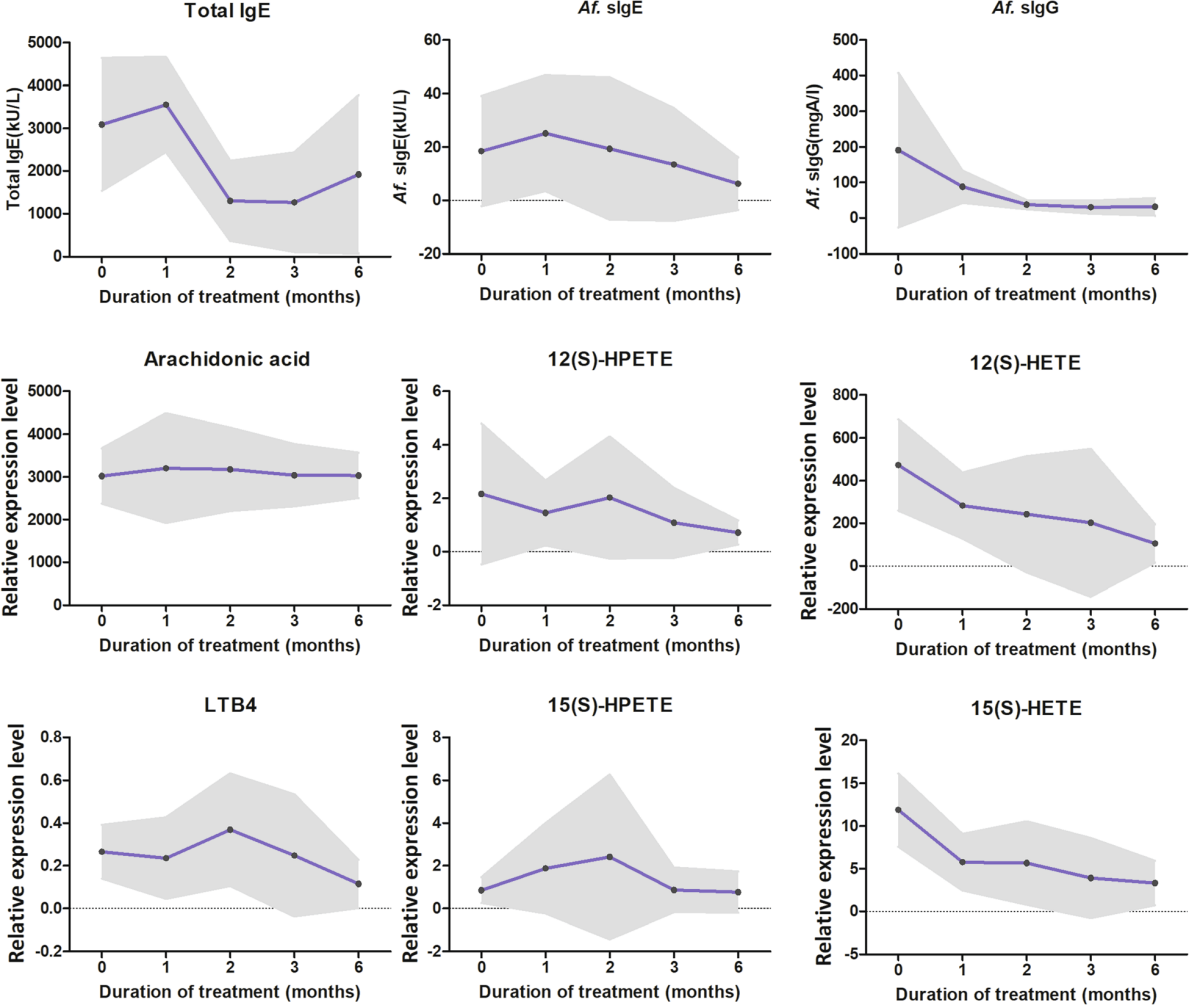
Trends of serum eicosanoids in ABPA patients during the treatment period. Serum samples of 11 ABPA patients were collected prior to and at 1, 2, 3 and 6 months after treatment. The points denote the mean value at each visit. The gray band show the standard deviation. *Af.*, *aspergillus fumigatus;* HPETE, hydroperoxyeicosatetraenoic acid; HETE, hydroxyeicosatetraenoic acid; LTB4, leukotriene B4

We further measured these metabolite levels during the treatment in ABPA patients. As shown in Fig. 3, the mean total IgE level at baseline was 3087 kU/L and decreased with medication. Similarly, serum *A. fumigatus s*IgE and *s*IgG levels also showed downward trends following treatment. The *A. fumigatus*-sIgG level gradually decreased from 191 mgA/L to 32 mgA/L. However, AA level did not change with treatment. It is worth noting that both 12(S)-HETE and 15(S)-HETE levels gradually decreased during the periods while their precursors 12(S)-HPETE and 15(S)-HPETE changed little. The other metabolite levels also change littler (data no shown).

## Discussion

The importance of lipid mediators as diagnostic markers and therapeutic targets in allergic diseases has been recognized and is evolving. However, little is known about their roles in ABPA. To our knowledge, this is the first report on serum lipid metabolite levels and the association of serum 12(S)-and 15(S)-HETE levels with the treatment in ABPA patients.

AA is oxidatively metabolized by the lipoxygenase (LOX) and cyclooxygenase (COX) pathways [16]. Previous studies have demonstrated that LOX-derived HETEs may be important in the pathogenesis of asthma and other allergic disease. In current study, we find that the levels of 4 kinds of HETEs in ABPA patients are significantly higher than that in healthy controls. We observed a higher level of 15(S)-HETE at baseline in ABPA patients and the level decreased with medication. It is reported that 15(S)-HETE is correlated with increasing asthma severity and high 15(S)-HETE level is a symbol of pro-inflammatory responses [17–19]. Moreover, 15(S)-HETE is generated by the direct conversion of AA by 15-LOX [20]. And the Th2 cytokines IL-4 and IL-13 both up-regulate 15-LOX. Maybe the higher inflammation reaction and Th2 bias in ABPA patients induced the higher 15(S)-HETE level in serum. Further research is needed to confirm our hypothesis. On the other hand, eosinophils were known to produce abundant 15(S)-HETE [21] and eosinophilia in asthma has been associated with 15-HETE [19]. This is in accord with our current results that the 15(S)-HETE positively correlated with peripheral blood eosinophils in ABPA.

In ABPA and asthma patients, 12(S)-HPETE and its downstream metabolite 12(S)-HETE both increased comparing with healthy controls. Moreover, 12(S)-HETE was the most abundant in serum compared with other lipid mediators. Same as 15(S)-HETE, 12(S)-HETE is reported to be involved in pro-inflammatory actions and produced by blood platelets and eosinophils [22–24]. Thus it is not difficult to explain 12(S)-HETE showed a positive correlation with peripheral blood eosinophils. Apart from 15(S)-HETE, 12(S)-HETE is the only one lipid mediator that its level decreased with treatment. However, it is worth mentioning that the usage and dose of oral corticosteroids could not influence the 12-HETE in Churg-Strauss syndrome [25], which suggest that 12(S)-HETE production might be steroid-insensitive. We speculated that there may be some factors influence the activation of 12-LOX and 15-LOX in ABPA and this may be a potential pathogenesis of this disease for the increasing sputum eosinophils. More research is needed to get more detailed information. A mouse model of ABPA indicated that *A. fumigatus* sensitization/challenge could cause an increase pulmonary gene expression of LOX-8, LOX-12e and LOX-15, which provided some theoretical basis for the increasing HETEs in ABPA patients [26].

In addition, some studies have reported that activated platelets could produce a broad range of AA-derived eicosanoids [27–29]. 12(S)-HETE is the earliest one to be demonstrated as the major platelet products that generated by 12-LOX and 11(S)-HETE and 15(S)-HETE were found to be major COX-1 products of platelets [28, 29]. In our study, levels of all these metabolites were significantly higher in patients with asthma or ABPA than in healthy controls. We think that platelets activation might play a role in the pathogenesis of asthma or ABPA. In fact, experimental evidence suggests that platelet activation by proinflammatory stimuli with subsequent platelet degranulation and chemokinesis are involved in all major features of asthma [30, 31]. However, we can only found one article about platelets and ABPA which reported that pro–platelet basic protein level was significantly increased in the ABPA [32]. But the mechanism is unclear. Our findings may prompt the role of platelet activation in ABPA disease from another aspect. But more experiments are needed to verify.

LTB4 is widely known as a potent chemoattractant for most subsets of leukocytes [33]. Previous study showed that levels of LTB4 in exhaled breath condensate from asthmatic patients are higher than nonasthmatic controls [34]. In our study, serum LTB4 level in ABPA patients significantly increased when compared with healthy controls. Moreover, LTB4 showed a positive correlation with sputum neutrophils in ABPA subjects. This is consistent with previous research that neutrophils can synthesize LTB4 by activating the 5-LOX signaling pathway in atherosclerosis [35]. Similar to LTB4, serum 5(S)-HETE in ABPA was significantly higher than that in healthy controls. However, 5(S)-HETE showed little effect on clinical outcome and the treatment of ABPA. This might be due to the little biological activity of 5(S)-HETE. It was reported that 5(S)-HETE can convert to 5-oxo-ETE, a potent chemoattractant for eosinophils [36], neutrophils [37] and monocytes [38]. Regretfully, we did not measure 5-oxo-ETE in this study.

In addition to AA metabolite, the metabolites derived from EPA and linoleic acid also involved in pathogenesis of ABPA. However, the role of these mediators in allergic diseases remain unclear. *A. fumigatus* sIgE is a characteristic diagnostic indicator of ABPA. Of the 36 metaboliters that have been measured, 12(S)-HPETE, 15(S)-HEPE and 12(S)-HEPE showed positive correlation with the *A. fumigatus* sIgE level. To our knowledge, there is no direct correlation between 15(S)-, 12(S)-HEPE and sIgE. But it is reported that 15-HEPE has an anti-allergic effect by inhibiting mast cell degranulation without affecting allergen sIgE production [39].15-HEPE may be involved in the pathogenesis of ABPA through this pathway. Moreover, 13(S)-HPODE and 9(S)-HPODE were negatively correlated with the percentage of sputum eosinophils. But more details about how they react need to be explored.

We recognize that there were some limitations in this study, such as the lack of samples from airway to confirm the direct action of these lipid mediators in ABPA patients. Moreover, the study may have been underpowered for the little group size. The results of our study were exploratory and need to be confirmed by further studies. Even so, these data provide pilot information for future power analysis. Further prospective studies are required to investigate the specific role of the lipid mediators in the pathogenesis of ABPA disease.

## Conclusions

In summary, our study demonstrates that metabolites of polyunsaturated fatty acid may participate in the pathogenesis of ABPA. Especially LOX-derived 15(S)-HETE and 12(S)-HETE from AA might be involved in ABPA. New ideas might be provided for the diagnosis, therapy and monitor of ABPA via targeting those specific LOX pathways.

## Data Availability

The datasets used and/or analysed during the current study are available from the corresponding author on reasonable request.

## Abbreviations

ABPA: Allergic bronchopulmonary aspergillosis
HC: Health control
PUFA: Polyunsaturated fatty acid
AA: Arachidonic acid
LOX: Lipoxygenase
COX: Cyclooxygenase
HETE: Hydroxyeicosatetraenoic acid
HEPE: Hydroxyeicosapentaenoic acid
HHTrE: Hydroxyheptadecatrienoic acid
HPETE: Hydroperoxyeicosatetraenoic acid
HPODE: Hydroperoxyoctadecadienoic acid
LT: Leukotriene
PG: Prostaglandin
Af: Aspergillus fumigatus
